# A study on resting-state functional near-infrared spectroscopy in patients with different outcomes of prolonged disorders of consciousness

**DOI:** 10.3389/fneur.2025.1533853

**Published:** 2025-06-02

**Authors:** Yanli Liang, Xin Liang, Yijiang Li, Chaowen Wang, Yinuo Bi, Yuan Xue, Wenyu Jiang

**Affiliations:** ^1^Department of Neurological Rehabilitation, Jiangbin Hospital of Guangxi Zhuang Autonomous Region, Nanning, China; ^2^Department of Rehabilitation Medicine, The First People's Hospital, Yulin, China; ^3^Data Science with Artificial Intelligence, University of Exeter, Exeter, United Kingdom; ^4^Cognitive Rehabilitation Center, Jiangbin Hospital of Guangxi Zhuang Autonomous Region, Nanning, China

**Keywords:** resting-state, functional near-infrared spectroscopy, prolonged disorders of consciousness, outcomes, functional connectivity

## Abstract

**Background:**

To explore the characteristics of resting-state functional connectivity in patients with different outcomes of prolonged disorders of consciousness (pDoC) by studying resting-state near-infrared imaging in patients with pDoC.

**Methods:**

60 patients with pDoC were processed with resting-state near-infrared imaging and divided into unresponsive wakefulness syndrome/vegetative state (UWS/VS) group, minimally conscious state (MCS) group and escape minimally conscious state (EMCS) group according to the post-treatment state of consciousness, to analyze the difference of resting-state functional connectivity in patients with different outcomes of patients with pDoC.

**Results:**

Functional connectivity (FC) between frontal lobe and left occipital lobe, frontal lobe and right occipital lobe, and left and right occipital lobes decreased in the UWS/VS group compared with the MCS group; functional connectivity between frontal lobe and left occipital lobe, frontal lobe and right occipital lobe, and left and right occipital lobes decreased in the UWS/VS group compared with the EMCS group; functional connectivity did not show any significant difference between the EMCS and MCS groups; and functional connectivity was more centralized in the MCS group and EMCS group.

**Conclusion:**

Different outcomes of patients with pDoC have different degrees of decline in functional connectivity between frontal lobe and occipital lobe and between occipital lobe, resting-state functional near-infrared spectroscopy has a certain reference significance for the prognosis of patients with pDoC, and it is helpful for exploring the exploration of the conscious residual brain areas.

## 1 Introduction

Over the past few decades, significant advances have been made in emergency procedures and techniques for the common causes of disorders of consciousness (DOC)-cardiac arrest, cerebral hemorrhage, massive cerebral infarction, and severe traumatic brain injury. However, the ensuing medical treatment of DOC patients from coma to recovery of consciousness is indeed a long and extremely costly process. Up to 40% of unresponsive brain-injured patients have their residual consciousness misjudged (1), leading to abandonment of subsequent treatment. Early identification or prediction of the prognosis and outcome of future awakenings in DOC patients is an appropriate pathway for rational treatment and rational control of healthcare costs.

According to the guidelines related to disorders of consciousness of the American Academy of Neurology and the implementation of the subcommittee system, the patient’s age, etiology, and duration of vegetative state are closely related to the recovery of consciousness (2). However, these are vague indicators, and in order to assess the value of treatment at an early stage, scholars have been trying to find more objective and accurate markers to assess the prognosis of patients with pDoC. In the study of consciousness in the brain, scientists have proposed the hypothesis of a “global neural workspace” based on a connectionist theoretical framework, and selective hypometabolism of the medial prefrontal cortex (mPFC), as the neural basis of consciousness, has been reported in a wide range of altered states of consciousness, such as sleep (3), drug-induced anesthesia (4), and acquired chronic pDoC states (5, 6). It can be used to assist in assessing the level of consciousness in DOC patients (7–9) and its functional connectivity strength is strongly associated with the prognosis of DOC patients (10). This suggests that enhanced functional connectivity of the mPFC predicts recovery of the neural network of consciousness. However, the recovery of the patient’s state of consciousness seems to be related not only to the internal cognitive network, but also to the external network of consciousness. For example, clinical means of awakening are often applied in terms of visual stimulation, and it has been observed that patients with earlier visual following, visual localization, and visual recognition seem to be more prone to awakening from the coma. Visual pursuit is considered to be one of the first signs that first appear during the recovery of consciousness (11). Studies have shown that moderate electroencephalography (EEG) frequencies dominated by alpha rhythms suggest a good prognosis for DOC patients (12). Some scholars have argued, based on their research, that the most representative *α*-rhythm is the occipital alpha rhythm from the visual cortex, which plays an important role in cognitive processes and sensory perception, and the occipital *α*-rhythm seems to reflect conscious perception and alertness in awake individuals (13–15). EEG studies have shown that loss of consciousness is associated with impaired information sharing over medium and long distances, reflecting the importance of long-distance cortical communication (16). Thus, long-range information transfer between the frontal-occipital lobes in the brains of DOC patients may reflect an internal orienting process of visual perception that is closely related to the patient’s awareness of the environment.

In recent years, techniques such as functional neuroimaging functional magnetic resonance imaging (fMRI) and positron emission tomography (PET) and electrophysiology (EEG) have played a role in making prognostic judgments in patients with disorders of consciousness. Rundgree et al. found that patients with impaired consciousness after cardiac arrest had a correspondingly higher rate of poor prognosis as the severity of amplitude-integrated EEG increased (17). Moreover, a multicenter cohort study applying resting-state functional magnetic resonance imaging found that connectivity within the default mode network (DMN) in the patient’s brain was highly correlated with the probability of recovery (18). PET imaging studies in comatose patients following hypoxic or traumatic brain injury have demonstrated that early inflammatory components are predominantly located in key cortical and subcortical brain structures that are thought to be involved in the emergence of consciousness (19). The above functional brain imaging techniques have expanded the means of diagnosis and evaluation in the field of DOC, however, they also have certain limitations. fMRI and PET equipment are expensive, have high spatial resolution, and operate in a large space, which does not allow for bedside measurements. EEG is easy to operate at the bedside, but is susceptible to the magneto-electric environment and has low spatial resolution.

Function near-infrared spectroscopy (fNIRS) has become one of the key technologies to study the neural mechanism of brain processing because of its portable, inexpensive and non-invasive advantages (20). Molteni et al. (21) used three stimulation modalities to detect residual functional brain activity in two MCS, with passive motor stimulation having the strongest response, somatosensory stimulation the next strongest, and active stimulation the weakest, and Kempny et al. (22) found that patients with MCS possessed more of the typical fNIRS response (elevated HbO2 with a concomitant decrease in HbR) compared to patients with vegetative state. In relation to the prefrontal cortex, fNIRS showed a significant advantage due to no hair in detecting the cognitive tasks like mental arithmetic, music imagery, emotion induction, etc. (23). Therefore, fNIRS is more valuable in detecting changes in frontal and occipital functional connectivity in patients with pDoC.

Based on the above, we hypothesized that utilizing the functional network performance of fNIRS between the frontal-occipital lobes may reveal resting-state functional connectivity patterns in the pDoC. To the best of our knowledge, there are no studies that provide a complete and comprehensive assessment of this critical fronto-occipital lobe connection in coma, although this information may be valuable in the development of assessment tools for the neurologic prognosis of comatose patients. In the current work, we aimed to measure the residual integrity of the FC of frontal-occipital brain structures in a cohort of mixed-case coma patients to gain insights into the neural mechanisms underlying the recovery of consciousness and to explore the residual brain regions of consciousness.

## 2 Materials and methods

### 2.1 Participants

Sixty patients with chronic consciousness disorder who were hospitalized in the Department of Neurological Rehabilitation of Guangxi Jiangbin Hospital from January 2022 to January 2023 were included in this study. Inclusion criteria: (1) the patients met the internationally established diagnostic criteria for UWS/VS as assessed by a specialized physician (24); (2) the duration of the consciousness disorder was greater than 28 days; (3) the preservation of the brainstem reflexes and the sleep–wake cycle; (4) All participants were not on drugs that affected hemodynamics for the first 3 days of the experiment; (5) the patients’ family members signed the informed consent form. Exclusion criteria: (1) patients with previous neuropsychiatric disorders; (2) use of central stimulants or sedative drugs 1 week before the experiment; (3) unstable vital signs; (4) hospitalization less than 1 month.

### 2.2 Experimental method

Subjects were admitted to the hospital for resting state fNIRS scanning, and a comprehensive treatment plan was adopted during hospitalization, which included blood pressure control, blood glucose control, anti-infection and comprehensive rehabilitation. All subjects received the same combination of treatments during their hospitalization. Our experiments were conducted in a quiet, light-free stimulating environment. After 1 month then 2 professionally trained doctors assessed the level of consciousness of the subjects according to the modified Coma Recovery Scale (CRS-R), and the subjects were divided into UWS/VS group, MCS group and EMCS group according to their different levels of consciousness.

### 2.3 fNIRS data collection and processing

**Figure 1:**
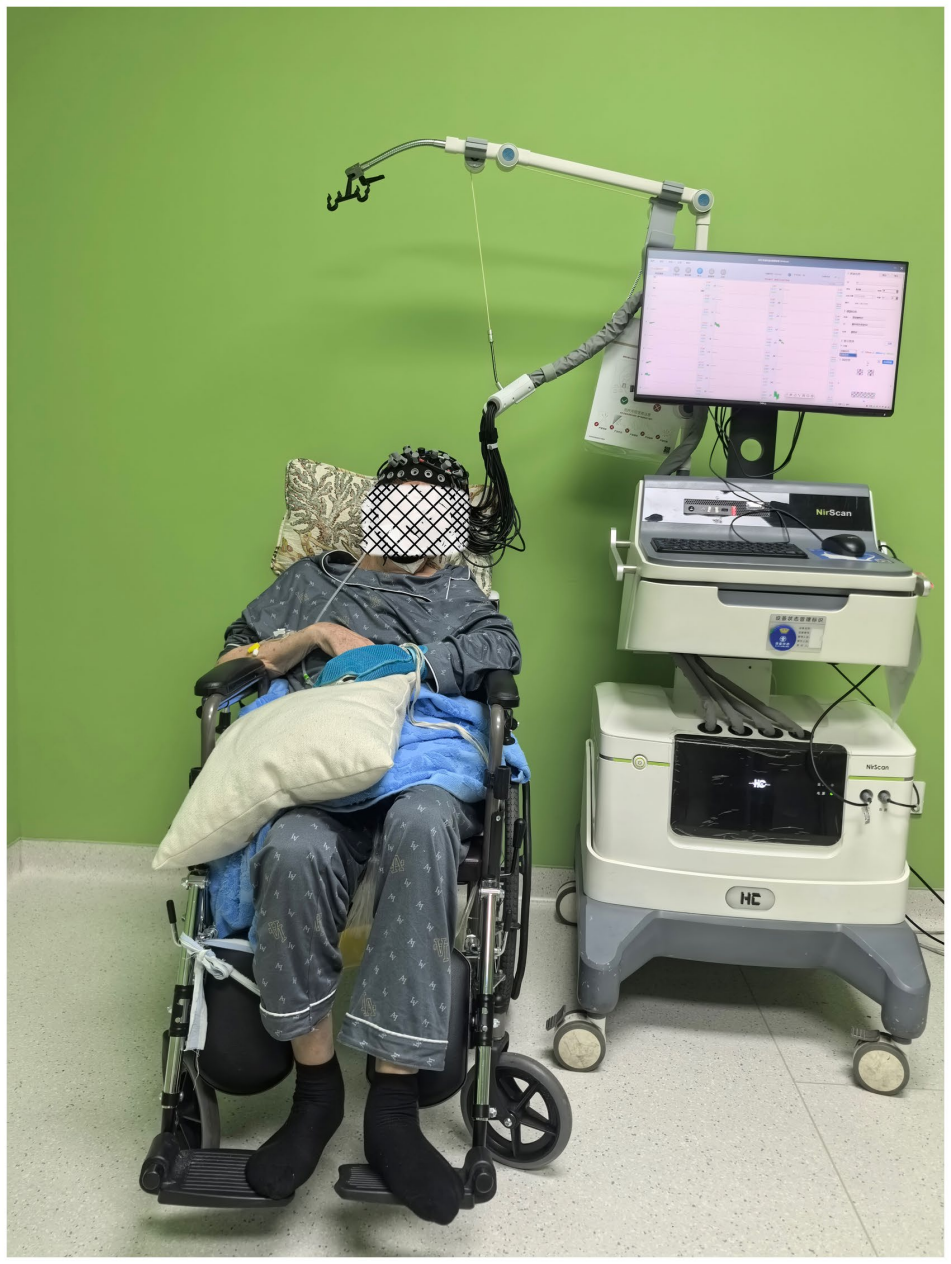
Functional near infrared acquisition device.

**Figure 2:**
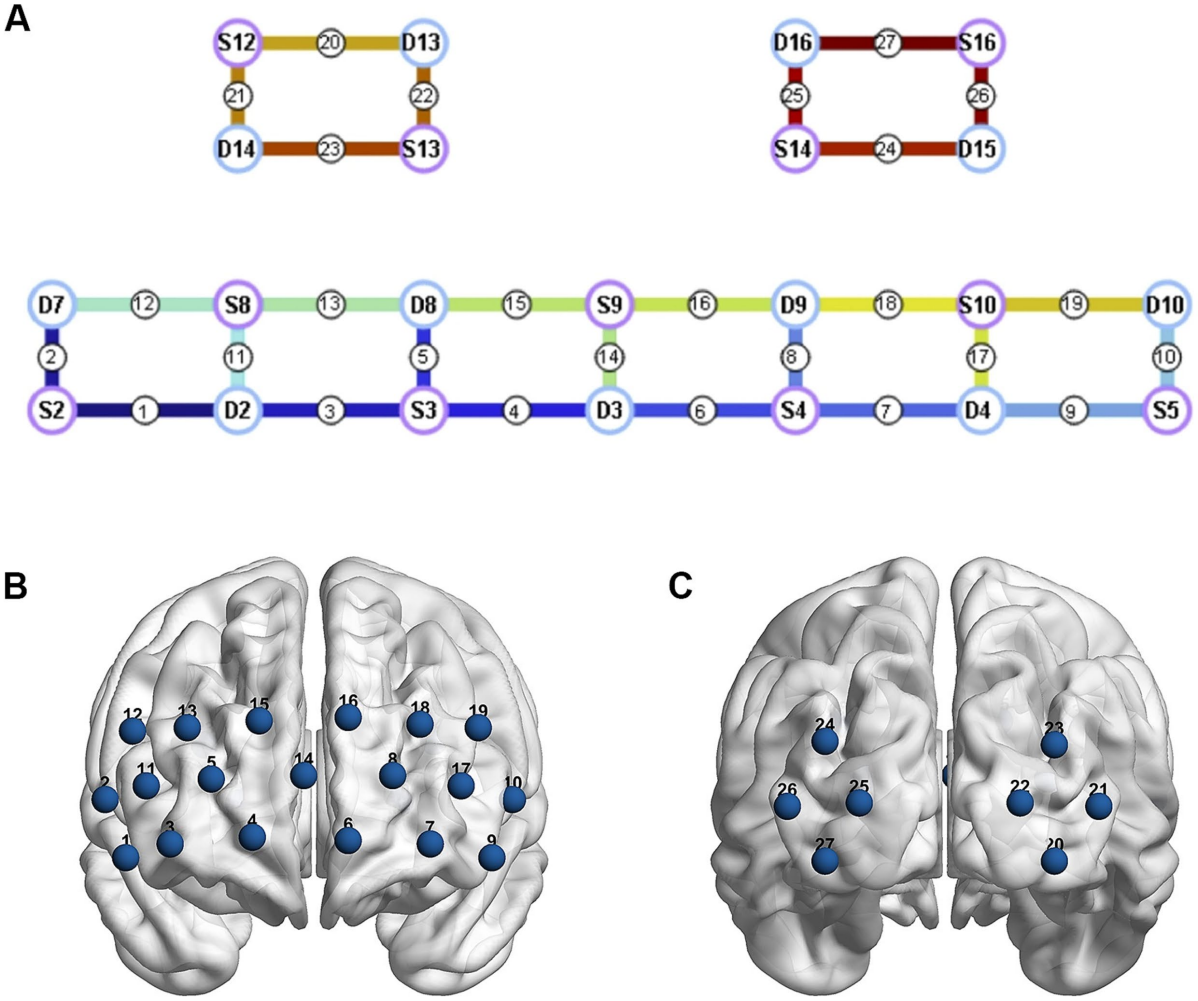
Channel arrangement of fNIRS brain functional imaging device. **(A)** The purple (S) and blue circles (D) represent the light source and the detector respectively, while the connecting lines between them represent the channels. **(B)** The layout diagram of 19 channels on the frontal lobe. **(C)** The layout diagram of 8 channels on the occipital lobe.

1. Resting-state fNIRS scanning: NirScan-6000C model fNIRS brain functional imaging device from Danyang Huichuang Medical Company (Figure 1) was used for resting-state fNIRS collection, with a total of 27 two-wavelength (730 nm, 850 nm) measurement channels in the bilateral brain, including 11 receivers and 11 light sources covering the frontal-occipital lobe region, with 19 channels (CH9~CH27) in the frontal lobe, and 8 channels (CH1–CH8) in the occipital lobe, the specific distribution and brain areas are shown in Figure 2 and Table 1, and all the signals were acquired at 12 Hz. Experimental data collection process time between 20 and 30 min.
2. fNIRS data processing: NirSpark software (HuiChuang, China) was used to analyze the fNIRS data. The steps were as follows: (1) correction for motion artifacts; (2) the initial light intensity data was converted to optical density; (3) removal of environmental and physiological noise with a bandpass filter of 0.01–0.20 Hz (25); (4) the filtered OD data was transmitted into the relative changes in the concentration of the HbO2 and HbR data based on the modified Beer–Lambert Law (26). Since HbO2 is a more reliable indicator of cortical blood flow changes (27) and exhibits a higher signal-to-noise ratio compared to HbR (28), it was selected for further analysis. (5) The Pearson’s correlation coefficient of the HbO2 concentration time series between each channel pair was calculated, and the coefficient was defined as the functional connectivity strength of the corresponding channel pair.

**Table 1:** The corresponding brain regions of 27 fNIRS channels.

Label of channel	Aal area	Percentage
CH1 (S2-D2)	Frontal_Mid_Orb_R	0.030612
	Frontal_Inf_Tri_R	0.13605
	Frontal_Inf_Orb_R	0.83333
CH2 (S2-D7)	Frontal_Inf_Oper_R	0.16667
	Frontal_Inf_Tri_R	0.83333
CH3 (S3-D2)	Frontal_Sup_R	0.0079681
	Frontal_Sup_Orb_R	0.099602
	Frontal_Mid_R	0.23506
	Frontal_Mid_Orb_R	0.65737
CH4 (S3-D3)	Frontal_Sup_R	0.33121
	Frontal_Sup_Orb_R	0.42994
	Frontal_Mid_Orb_R	0.031847
	Frontal_Sup_Medial_R	0.1051
	Frontal_Mid_Orb_R	0.10191
CH5 (S3-D8)	Frontal_Sup_R	0.83755
	Frontal_Mid_R	0.16245
CH6 (S4-D3)	Frontal_Sup_L	0.17822
	Frontal_Sup_Orb_L	0.25413
	Frontal_Sup_Medial_L	0.21452
	Frontal_Mid_Orb_L	0.35314
CH7 (S4-D4)	Frontal_Sup_L	0.26354
	Frontal_Sup_Orb_L	0.12274
	Frontal_Mid_L	0.072202
	Frontal_Mid_Orb_L	0.54152
CH8 (S4-D9)	Frontal_Sup_L	0.97122
	Frontal_Mid_L	0.028777
CH9 (S5-D4)	Frontal_Mid_Orb_L	0.092857
	Frontal_Inf_Tri_L	0.12143
	Frontal_Inf_Orb_L	0.78571
CH10 (S5-D10)	Frontal_Inf_Oper_L	0.022222
	Frontal_Inf_Tri_L	0.97778
CH11 (S8-D2)	Frontal_Mid_R	0.95094
	Frontal_Inf_Tri_R	0.049057
CH12 (S8-D7)	Frontal_Mid_R	0.5
	Frontal_Inf_Tri_R	0.5
CH13 (S8-D8)	Frontal_Mid_R	1
CH14 (S9-D3)	Frontal_Sup_Medial_L	0.48013
	Frontal_Sup_Medial_R	0.51987
CH15 (S9-D8)	Frontal_Sup_R	0.55303
	Frontal_Mid_R	0.098485
	Frontal_Sup_Medial_R	0.34848
CH16 (S9-D9)	Frontal_Sup_L	0.4359
	Frontal_Sup_Medial_L	0.5641
CH17 (S10-D4)	Frontal_Mid_L	0.88142
	Frontal_Inf_Tri_L	0.11858
CH18 (S10-D9)	Frontal_Sup_L	0.23377
	Frontal_Mid_L	0.76623
CH19 (S10-D10)	Frontal_Mid_L	0.65504
	Frontal_Inf_Tri_L	0.34496
CH20 (S12-D13)	Lingual_R	0.20599
	Occipital_Mid_R	0.10112
	Occipital_Inf_R	0.69288
CH21 (S12-D14)	Occipital_Mid_R	0.88618
	Occipital_Inf_R	0.11382
CH22 (S13-D13)	Calcarine_R	0.26756
	Cuneus_R	0.10368
	Lingual_R	0.0033445
	Occipital_Sup_R	0.54181
	Occipital_Mid_R	0.076923
	Occipital_Inf_R	0.006689
CH23 (S13-D14)	Occipital_Sup_R	0.42697
	Occipital_Mid_R	0.57303
CH24 (S14-D15)	Occipital_Sup_L	0.12044
	Occipital_Mid_L	0.87956
CH25 (S14-D16)	Occipital_Sup_L	0.052265
	Occipital_Mid_L	0.94774
CH26 (S16-D15)	Occipital_Mid_L	1
CH27 (S16-D16)	Lingual_L	0.19615
	Occipital_Mid_L	0.58846

### 2.4 Statistical analysis

The gender differences of the three groups were compared using the chi-square test, and the age, education level, and disease duration of the three groups were analyzed using one-way ANOVA, with *p* < 0.05 being statistically significant; the fNIRS data were statistically analyzed using NirSpark software, and two-way ANOVA was used to compare the effects of different groups and types of connectivity (frontal lobe and left occipital lobe, frontal lobe and right occipital lobe, and left and right occipital lobe) on the functional connectivity. Comparisons of functional connectivity between connectivity channels were analyzed by one-way ANOVA with false discovery rate (FDR)-corrected *p* < 0.05.

## 3 Results

### 3.1 Comparison of general information

The differences between the three groups in terms of age, gender, education level, and disease duration were not significant (Table 2). It shows that there is no statistical significance between the general information of the subjects in the three groups and they are comparable.

**Table 2:** Demographic data.

Characteristics	UWS/VS group	MCS group	EMCS group	*p*-value
Gender (M/F)	17/11	9/8	8/7	0.839
Age (years)	61.05 ± 11.31	64.15 ± 6.81	59.35 ± 7.91	0.232
Education (years)	10.35 ± 2.60	10.40 ± 2.58	10.30 ± 2.30	0.992
Disease duration (months)	6.30 ± 5.52	7.60 ± 6.68	6.50 ± 5.68	0.635

### 3.2 Effects of different groups and connection types on functional connectivity

Functional connectivity between the frontal lobe and the left occipital lobe, the frontal lobe and the right occipital lobe, and the right and left occipital lobes decreased in the UWS/VS group when compared with the MCS group (*p* < 0.05). Functional connectivity between the frontal lobe and the left occipital lobe, the frontal lobe and the right occipital lobe, and the right and left occipital lobes decreased in the UWS/VS group compared with the EMCS group (*p* < 0.05). Compared with the EMCS group, no significant differences were observed in the functional connectivity between the frontal lobe and the left occipital lobe, the frontal lobe and the right occipital lobe, and the right and left occipital lobes in the MCS group (*p* > 0.05) (Table 3). The functional strengths of the different connection types in the three groups are shown in Table 4.

**Table 3:** Results of functional connection comparison among three groups with different connection types.

Groups	Connection type	T-Value	*p*-value
UWS/VS group VS MCS group	Frontal lobe and left occipital lobe	−3.7413	0.0034
UWS/VS group VS MCS group	Frontal lobe and right occipital lobe	−2.8638	0.0080
UWS/VS group VS MCS group	Left and right occipital lobes	−3.3691	0.0034
UWS/VS group VS EMCS group	Frontal lobe and left occipital lobe	−6.3753	9.9295e-06
UWS/VS group VS EMCS group	Frontal lobe and right occipital lobe	−5.3242	4.1184e-05
UWS/VS group VS EMCS group	Left and right occipital lobes	−3.9125	0.0010
MCS group VS EMCS group	Frontal lobe and left occipital lobe	−1.8881	0.1075
MCS group VS EMCS group	Frontal lobe and right occipital lobe	−2.6459	0.0533
MCS group VS EMCS group	Left and right occipital lobes	−1.1170	0.2755

**Table 4:** Functional strength of three groups different connection types.

Connection type	UWS/VS group	MCS group	EMCS group
Frontal lobe and left occipital lobe	0.0689 ± 0.0138	0.2654 ± 0.1267	0.3888 ± 0.1132
Frontal lobe and right occipital lobe	0.0745 ± 0.0205	0.2013 ± 0.1012	0.3657 ± 0.1445
Left and right occipital lobes	0.0892 ± 0.0256	0.2906 ± 0.1035	0.3928 ± 0.1436

### 3.3 Functional connectivity diagrams of the three groups

**Figure 3:**
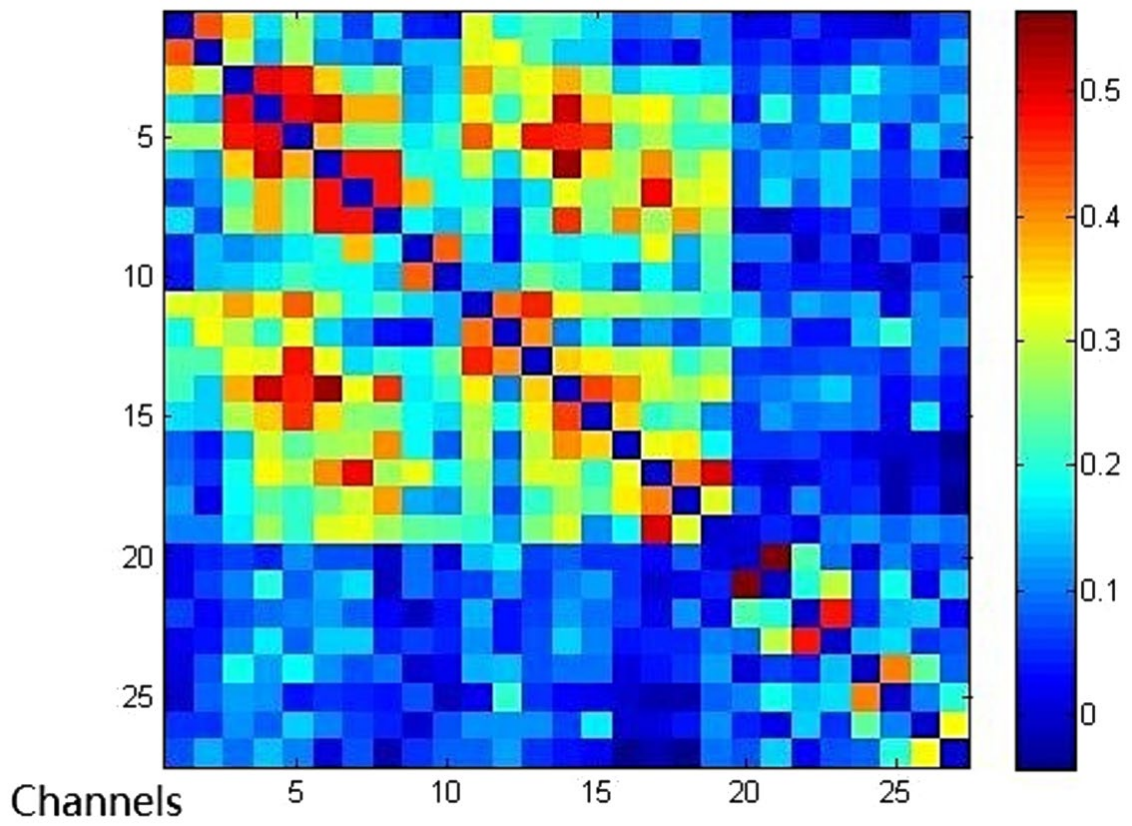
Functional connection of each channel in UWS/VS group. Connectivity matrix of 27 channels in UWS/VS group.

**Figure 4:**
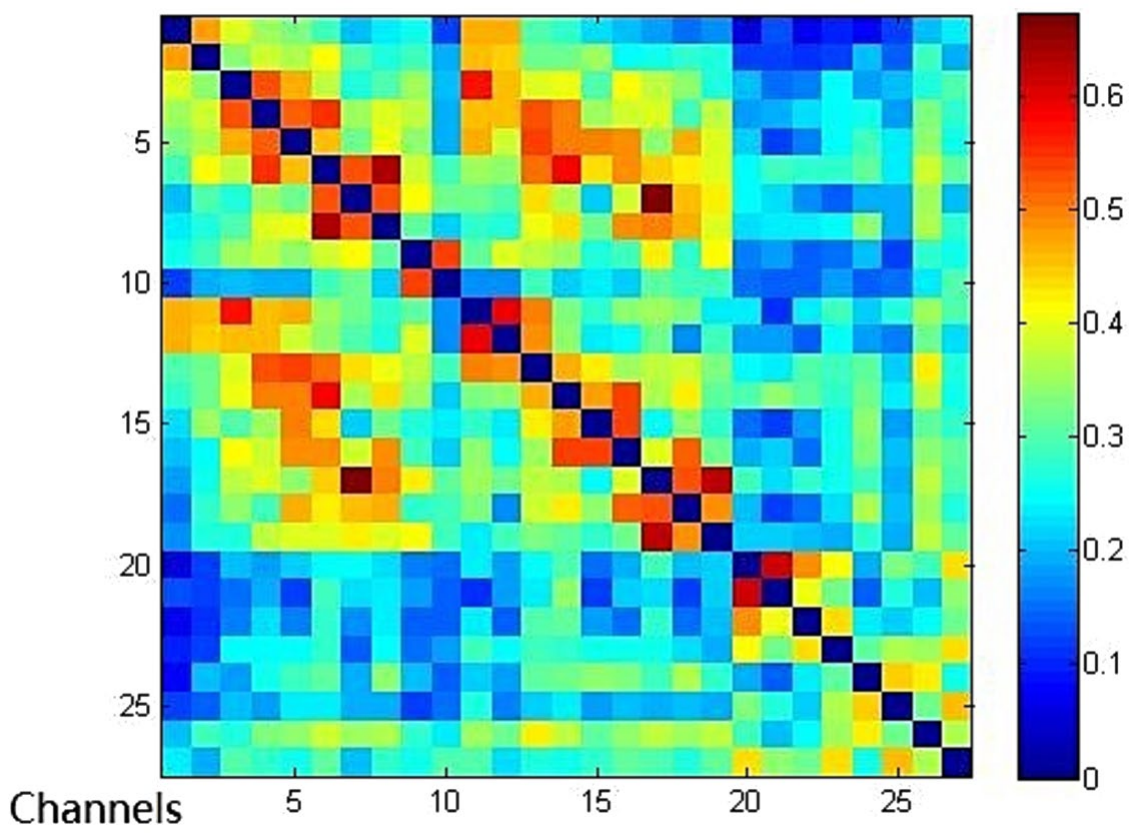
Functional connection of each channel in MCS group. Connectivity matrix of 27 channels in MCS group.

**Figure 5:**
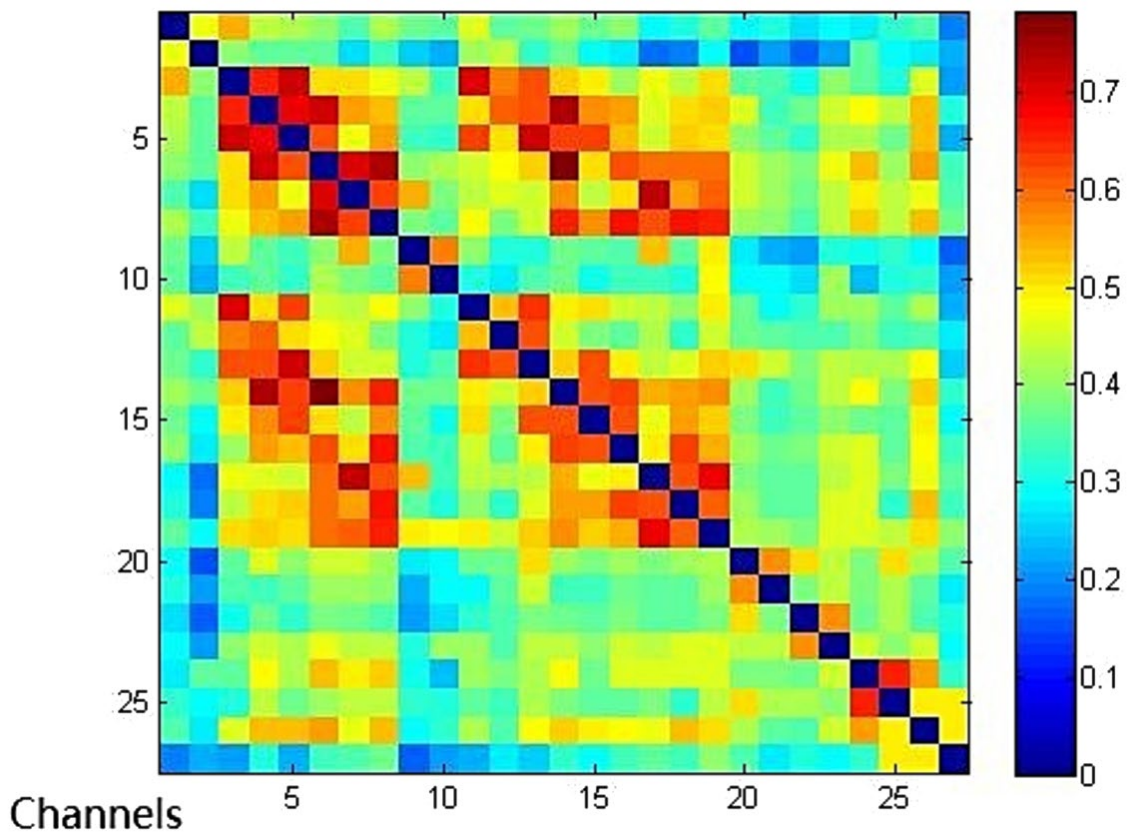
Functional connection of each channel in EMCS group. Connectivity matrix of 27 channels in EMCS group.

**Figure 6:**
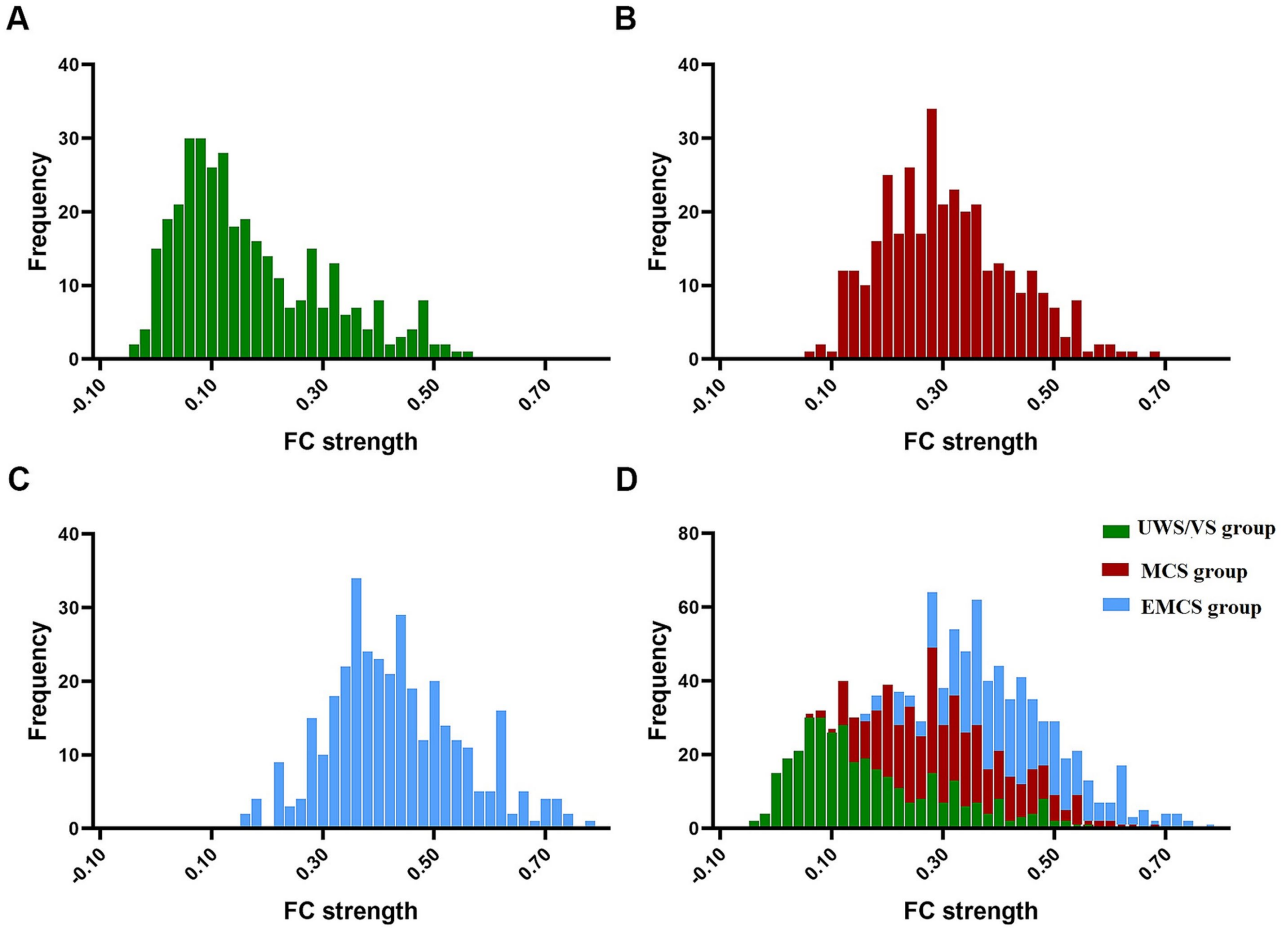
Three groups of average functional connection strength distribution. **(A)** Average functional connection strength distribution in UWS/VS group. **(B)** Average functional connection strength distribution in MCS group. **(C)** Average functional connection strength distribution in EMCS group. **(D)** Average functional connection strength distribution in three groups.

Compared with the EMCS group, the channel connectivity strength of the MCS group and the UWS/VS group showed a decreasing trend in overall (Figures 3–5). The functional connectivity strengths of the MCS group and the EMCS group were large, distributed and more centralized, whereas the functional connectivity strengths of the UWS/VS group were small and discrete, and the histograms of mean functional connectivity strengths of the three groups are shown in Figure 6.

## 4 Discussion

In this study, we found that the UWS/VS group showed a significant decrease in functional connectivity between the frontal lobe and the left occipital lobe, the frontal lobe and the right occipital lobe, and the right and left occipital lobes compared with the EMCS group; however, no significant functional connectivity differences were demonstrated compared with the MCS group. There were significant differences in functional connectivity between the frontal and left occipital lobes, frontal and right occipital lobes, and left and right occipital lobes between the MCS and UWS/VS groups. Patients with different outcomes of pDoC had different degrees of functional connectivity alterations in resting-state fNIRS, which may be the direction of our future research on patients with pDoC prognosis.

We chose two brain regions, the frontal and occipital lobes, for the study of patients with disorders of consciousness, and both showed that patients with disorders of consciousness have differences in both inter- and intra-brain functional connectivity between these two brain regions, and that this difference may be evidence for us to determine prognosis. Decreased functional connectivity may be associated with reduced efficiency of information transfer between brain regions. It was shown that the prefrontal cortex, an important node of the consciousness pathway, exhibited significant functional connectivity abnormalities in patients with UWS/VS and MCS, which may reflect the critical role of the prefrontal lobe in the recovery of consciousness (29). Thibaut et al. (30) reported for the first time that tDCS stimulation of the left dorsolateral prefrontal cortical area showed improvement in consciousness in some MCS patients, confirming that the left dorsolateral prefrontal cortical area is a key node in the network of disorders of consciousness. In an experiment by Silva et al. (6), a significant correlation between patients’ CRS-R scores and posterior cingulate-medial prefrontal cortex activity in the resting state, in particular a reduction in functional connectivity between the medial prefrontal cortex and posterior cingulate cortex, predicted poor outcome in patients with disorders of consciousness. All of these studies demonstrate the importance of the frontal lobe in brain function in patients with disorders of consciousness.

Most previous studies on disorders of consciousness have ignored the role played by the occipital lobe in disorders of consciousness, but this paper found that the occipital lobe has different degrees of decreased functional connectivity in patients with disorders of consciousness. An fNIRS study of patients with impaired consciousness found that functional connectivity between prefrontal and occipital regions was significantly elevated in patients after transspinal stimulation, which demonstrates that increased connectivity strength between prefrontal and occipital regions is associated with improved consciousness (31). The results of the present study also support that the functional connectivity between the occipital lobes and between the occipital lobes and frontal lobes decreased in patients with minimally conscious state and persistent vegetative state compared to awake patients. Daniel Golkowski et al. (32) simultaneously applied fMRI, deoxyglucose positron emission tomography (FDG-PET), and EEG to assess the prognosis of patients with disorders of consciousness, and found that glucose metabolism in the occipital lobe was significantly higher in patients with MCS than in patients with VS as measured by FDG-PET, suggesting that the occipital lobe plays an important role in the recovery of consciousness, similar to the results in this study.

The present study did not observe the differences in functional connectivity between the MCS and EMCS groups in the three connectivity modality, which may be due to the fact that some of the MCS patients were very close to the EMCS group in the level of the conscious state and the FNIRS could not capture the small differences in the brain. Although the overall level of consciousness is higher in MCS patients than in UWS/VS group, both may have a similar degree of impairment of underlying network connections in the resting state due to similar widespread cortical damage (33). On the other hand, it is possible that the sample size limitation resulted in the failure to statistically reflect the differences between the two groups.

The tendency for functional connectivity strength to decrease with the gradient of level of consciousness (EMCS>MCS > UWS/VS) suggests that resting-state fNIRS metrics may serve as a quantitative complementary tool for consciousness assessment. Compared to clinical behavioral scales that are susceptible to motor functional limitations, functional connectivity parameters may be more sensitive to underlying neurological remodeling.

## 5 Limitations

The present study still has some limitations, as the lack of sample size led to the failure of subgrouping according to the etiology to observe the differences in brain functional connectivity between different causes of disorders of consciousness. In addition, due to the limited hospitalization period of some patients, it was not possible to follow up the fNIRS data for each group.

## 6 Conclusion

Overall, fNIRS can be used in the future as a new functional brain imaging technique, which is expected to assess the prognosis of patients with disorders of consciousness by detecting the strength of connectivity in functional brain regions. The resting-state-based fNIRS data increase the understanding of neuroimaging in patients with chronic disorders of consciousness, and the results of this study provide a theoretical basis for neuroimaging to study disease prognosis in patients with chronic disorders of consciousness.

## Data Availability

The original contributions presented in the study are included in the article/supplementary material, further inquiries can be directed to the corresponding author.
