# Correction: A study on resting-state functional near-infrared spectroscopy in patients with different outcomes of prolonged disorders of consciousness

**DOI:** 10.3389/fneur.2025.1642987

**Published:** 2025-06-27

**Authors:** Yanli Liang, Xin Liang, Yijiang Li, Chaowen Wang, Yinuo Bi, Yuan Xue, Wenyu Jiang

**Affiliations:** ^1^Department of Neurological Rehabilitation, Jiangbin Hospital of Guangxi Zhuang Autonomous Region, Nanning, China; ^2^Department of Rehabilitation Medicine, The First People's Hospital, Yulin, China; ^3^Data Science with Artificial Intelligence, University of Exeter, Exeter, United Kingdom; ^4^Cognitive Rehabilitation Center, Jiangbin Hospital of Guangxi Zhuang Autonomous Region, Nanning, China

**Keywords:** resting-state, functional near-infrared spectroscopy, prolonged disorders of consciousness, outcomes, functional connectivity

The Funding statement of the original article was corrected from: “The study was supported by the grant from the self-funded project by the Health Commission of Guangxi Zhuang Autonomous Region (grant no. Z220170172).” to “The study was supported by the grant from the Qingxiu District Science and Technology Program Project of Nanning (Grant No. 2018037).”

The original version of this article has been updated.

